# Supplementary material for: Association between diabetes mellitus and impaired single-leg stance in patients with chronic liver disease: A cross-sectional study
Source: PLoS One. 2026 Mar 24;21(3):e0345407. doi: 10.1371/journal.pone.0345407 (PMC13012516; doi:10.1371/journal.pone.0345407)
Supplement: S1 Table — (DOCX) [file pone.0345407.s001.docx]

**S1 Table. Characteristics of the patients included in this study (N = 152)**

| **Characteristics** | **Values** |
| --- | --- |
| **Age (years)** | 68 (61–73) |
| **Sex (female)** | 64 (42) |
| **Height (cm)** | 160.5 (153.9–166.9) |
| **Weight (kg)** | 63.5 (57.6–75.3) |
| **BMI (kg/m^2^)** | 25.1 (22.4–29.4) |
| **> 25** | 77 (51) |
| **Blood data** |  |
| **HGB (g/dL)** | 13.5 (12.3–14.9) |
| **PLT (10^4^/μL)** | 15.9 (10.9–21.3) |
| **T-BIL (mg/dL)** | 0.9 (0.7–1.2) |
| **AST (IU/L)** | 32 (23–48) |
| **ALT (IU/L)** | 28 (19–52) |
| **ALB (g/dL)** | 4.2 (3.7–4.5) |
| **PT (%)** | 88 (76–97) |
| **NH_3_ (μmol/L)** | 36 (28–49) |
| **HbA1c (%)** | 6.8 (6.1–7.7) |
| **TG (mg/dL)** | 111 (84–161) |
| **UA (mg/dL)** | 5.4 (4.7–6.3) |
| **Etiology (ALD/HBV/HCV/MASLD/other)** |  |
| **ALD** | 17 (11) |
| **HBV** | 18 (12) |
| **HCV** | 58 (38) |
| **MASLD** | 49 (32) |
| **Others** | 10 (7) |
| **LC** | 14 (9) |
| **HCC** | 32 (9) |
| **Encephalopathy** | 1 (1) |
| **Ascites** | 12 (8) |
| **Child–Pugh score** | 5 (5–6) |
| **Uncompensated cirrhosis; Child–Pugh score ≥ 7** | 24 (16) |
| **Child–Pugh classification** |  |
| **A** | 127 (83) |
| **B** | 18 (12) |
| **C** | 7 (5) |
| **FIB**-**4 index** | 2.65 (1.69–4.28) |
| **APRI** | 0.76 (0.46–1.30) |
| **ECW/TBW** | 0.39 (0.384–0.395) |
| **SMI (kg/m^2^)** | 7.10 (6.40–7.76) |
| **Male < 7.0, Female < 5.7** | 41 (27) |
| **ASM/weight (%)** | 27.4 (24.0–31.3) |
| **ASM/BMI (kg/kg/m^2^)** | 0.71 (0.57–0.85) |
| **Upper limb muscle mass (kg/m^2^)** | 1.90 ± 0.43 |
| **KEF/weight (kgf/kg)** | 0.51 ± 0.16 |
| **SLST (s), Mean (Both legs)** | 28.3 (9.5–57.4) |
| **< 5** | 23 (15) |
| **SLST (s), Max (Either leg)** | 36.9 (10.9–60) |
| **< 5** | 16 (11) |
| **Grip strength (kg)** | 26.3 (19.9–33.6) |
| **Male < 28, Female < 18** | 48 (32) |
| **Gait speed (m/s)** | 1.12 ± 0.24 |
| **<1.0** | 41 (27) |
| **Body fat percentage (%)** | 31.17 ± 9.48 |
| **Male ≥ 27, Female ≥ 38** | 72 (47) |
| **Complications** |  |
| **DM** | 118 (78) |
| **HT** | 80 (53) |
| **DL** | 38 (25) |
| **Sarcopenia by 2019 AWGS** | 23 (15) |
| **Sarcopenia by JSH, 2^nd^ edition** | 21 (14) |

Continuous variables are presented as mean ± standard deviation or median (interquartile range). Categorical variables are presented as numbers (percentages). BMI, body mass index; HGB, hemoglobin; PLT, platelet; T-BIL, total bilirubin; AST, aspartate aminotransferase; ALT, alanine aminotransferase; ALB, albumin; PT, prothrombin time; NH₃, ammonia; HbA1c, hemoglobin A1c; TG, triglycerides; UA, uric acid; HBV, hepatitis B virus; HCV, hepatitis C virus; ALD, alcohol-associated liver disease; MASLD, metabolic dysfunction-associated steatotic liver disease; LC, liver cirrhosis; HCC, hepatocellular carcinoma; FIB-4, Fibrosis-4; APRI, AST-to-platelet ratio index; ECW/TBW, extracellular water-to-total body water ratio; SMI, skeletal muscle mass index; ASM, appendicular skeletal muscle mass; KEF, knee extension force; SLST, single-leg stance test; DM, diabetes mellitus; HT, hypertension; DL, dyslipidemia; AWGS, Asian Working Group for Sarcopenia; JSH, Japanese Society of Hepatology.
