# Supplementary material for: Association between diabetes mellitus and impaired single-leg stance in patients with chronic liver disease: A cross-sectional study
Source: PLoS One. 2026 Mar 24;21(3):e0345407. doi: 10.1371/journal.pone.0345407 (PMC13012516; doi:10.1371/journal.pone.0345407)
Supplement: S2 Table — (DOCX) [file pone.0345407.s002.docx]

**S2 Table.** **Comparison of background characteristics between patients with and without DM among those with CLD (N = 152)**

|  | **Patients with CLD (N = 152)** | |  |  |
| --- | --- | --- | --- | --- |
| **Characteristics** | **Non-DM (n = 34)** | **DM (n = 118)** | **p-value** | **Effect size** |
| **Age (years)** | 63 (56–71) | 68 (63–73) | 0.005** | 0.23 (*r*) |
| **Sex (male/female)** | 23 (68) / 11 (32) | 65 (55) / 53 (45) | 0.238 | 0.11 (*V*) |
| **Height (cm)** | 163.6 (154.2–169.1) | 159.9 (153.6–166.2) | 0.188 | 0.11 (*r*) |
| **Weight (kg)** | 67.1 (62.3–86.9) | 63.0 (55.5–72.9) | 0.023* | 0.18 (*r*) |
| **BMI (kg/m^2^)** | 27.1 (24.3–30.6) | 24.7 (22.0–28.8) | 0.025* | 0.18 (*r*) |
| **> 25** | 21 (62) | 56 (48) | 0.174 | 0.12 (*V*) |
| **Body fat percentage (%)** | 32.8 ± 9.2 | 30.7 ± 9.5 | 0.267 | 0.22 (*d*) |
| **Male ≥ 27, Female ≥ 38** | 17 (50) | 66 (47) | 0.846 | 0.03 (*V*) |
| **Complications** |  |  |  |  |
| **HT** | 16 (47) | 64 (54) | 0.559 | 0.06 (*V*) |
| **DL** | 8 (24) | 30 (25) | 1.000 | 0.02 (*V*) |
| **Blood data** |  |  |  |  |
| **HGB (g/dL)** | 13.9 (13.0–15.0) | 13.5 (12.0–14.8) | 0.145 | 0.12 (*r*) |
| **PLT (10^4^/μL)** | 16.2 (11.1–22.2) | 15.9 (10.7–20.1) | 0.823 | 0.02 (*r*) |
| **T-BIL (mg/dL)** | 0.9 (0.5–1.3) | 0.9 (0.7–1.2) | 0.508 | 0.05 (*r*) |
| **AST (IU/L)** | 42 (28–61) | 29 (22–46) | 0.004** | 0.23 (*r*) |
| **ALT (IU/L)** | 42 (27–75) | 26 (18–47) | 0.001** | 0.27 (*r*) |
| **ALB (g/dL)** | 4.2 (3.7–4.5) | 4.3 (3.7–4.5) | 0.804 | 0.02 (*r*) |
| **PT (%)** | 84 (67–96) | 89 (77–98) | 0.301 | 0.08 (*r*) |
| **NH_3_ (μmol/L)** | 39 (32–61) | 35 (27–49) | 0.084 | 0.14 (*r*) |
| **HbA1c (%)** | 5.8 (5.4–6.1) | 7.2 (6.6–8.0) | <0.001** | 0.59 (*r*) |
| **TG (mg/dL)** | 143 (93–176) | 106 (81–160) | 0.153 | 0.12 (*r*) |
| **UA (mg/dL)** | 286 (220–352) | 301 (217–359) | 0.913 | 0.01 (*r*) |
| **Etiology (ALD/HBV/HCV/MASLD/other)** |  |  | 0.633 | 0.13 (*V*) |
| **ALD** | 3 (9) | 14 (12) |  |  |
| **HBV** | 4 (12) | 14 (12) |  |  |
| **HCV** | 14 (41) | 44 (37) |  |  |
| **MASLD** | 9 (26) | 40 (34) |  |  |
| **Others** | 4 (12) | 6 (5) |  |  |
| **LC** | 2 (6) | 12 (10) | 0.737 | 0.06 (*V*) |
| **HCC** | 6 (18) | 26 (22) | 0.642 | 0.04 (*V*) |
| **Encephalopathy** | 0 (0) | 1 (1) | 1.000 | 0.04 (*V*) |
| **Ascites** | 2 (6) | 10 (8) | 1.000 | 0.04 (*V*) |
| **Child–Pugh score** | 5(5–6) | 5(5–6) | 0.862 | 0.01 (*r*) |
| **Uncompensated cirrhosis; Child–Pugh score ≥ 7** | 7 (21) | 17 (14) | 0.425 | 0.07 (*V*) |
| **Child–Pugh classification** |  |  | 0.760 | 0.06 (*V*) |
| **A** | 27 (79) | 100 (85) |  |  |
| **B** | 5 (15) | 13 (11) |  |  |
| **C** | 2 (6) | 5 (4) |  |  |
| **FIB-4 index** | 2.88 (1.31–3.83) | 2.63 (1.72–4.46) | 1.000 | 0.00 (*r*) |
| **APRI** | 1.08 (0.51–1.39) | 0.75 (0.44–1.15) | 0.068 | 0.15 (*r*) |

Continuous variables are presented as mean ± standard deviation or median (interquartile range), as appropriate. Categorical variables are presented as numbers (percentages). Effect sizes are interpreted as follows: Cohen’s *d* (0.2–0.5 = small, 0.5–0.8 = medium, and ≥ 0.8 = large); *r* and Cramer’s *V* (0.1–0.3 = small, 0.3–0.5 = medium, and ≥ 0.5 = large). *p < 0.05, **p < 0.01. CLD, chronic liver disease; DM, diabetes mellitus; BMI, body mass index; HT, hypertension; DL, dyslipidemia; HGB, hemoglobin; PLT, platelet; T-BIL, total bilirubin; AST, aspartate aminotransferase; ALT, alanine aminotransferase; ALB, albumin; PT, prothrombin time; NH₃, ammonia; HbA1c, hemoglobin A1c; TG, triglycerides; UA, uric acid; ALD, alcohol-associated liver disease; HBV, hepatitis B virus; HCV, hepatitis C virus; MASLD, metabolic dysfunction-associated steatotic liver disease; LC, liver cirrhosis; HCC, hepatocellular carcinoma; FIB4, Fibrosis-4; APRI, AST-to-platelet ratio index.
