# Supplementary material for: Association between diabetes mellitus and impaired single-leg stance in patients with chronic liver disease: A cross-sectional study
Source: PLoS One. 2026 Mar 24;21(3):e0345407. doi: 10.1371/journal.pone.0345407 (PMC13012516; doi:10.1371/journal.pone.0345407)
Supplement: Attachment — Submitted filename: Response to reviewers.docx [file pone.0345407.s004.docx]

May 28, 2025

Dr. Bisher Sawaf

Academic Editor

*PLOS ONE*

Dear Editor,

Thank you for your consideration of our manuscript previously titled *“Chronic liver disease complicated by diabetes mellitus is associated with impaired single-leg standing in patients: a cross-sectional study”* (Manuscript ID: [PONE-D-25-15349], EMID: 7be85993d2e2226f).

We have carefully revised the manuscript in accordance with the editors’ and reviewers’ suggestions. A point-by-point response to each comment is provided below. All changes in the revised manuscript are highlighted in red font. We sincerely hope that our responses and revisions have satisfactorily addressed all concerns, and that the manuscript is now suitable for publication in *PLOS ONE*.

Please note that we have updated the manuscript title to replace *“Chronic liver disease complicated by diabetes mellitus is associated with impaired single-leg standing in patients: a cross-sectional study”* with the more standard and academically appropriate title *“Association between diabetes mellitus and impaired single-leg stance in patients with chronic liver disease: a cross-sectional study.”*

The funders had no role in study design, data collection and analysis, decision to publish, or preparation of the manuscript.

Additionally, we would like to request an update to the second affiliation of the first author, Kenichi Fudeyasu. The corrected affiliation is:
**^2^ Department of Rehabilitation Medicine, Osaka Medical and Pharmaceutical University Hospital, Osaka, Japan.**

Please ensure this correction is reflected in the author list and manuscript metadata upon acceptance.

We greatly appreciate the opportunity to revise our work and look forward to your response.

Best regards,

Makoto Asaeda

Department of Rehabilitation Medicine, Hiroshima University Hospital

1-2-3 Kasumi, Minami-ku, Hiroshima 734-8551, Japan TEL: +81-82-257-5566

E-mail: [asaedam@hiroshima-u.ac.jp](mailto:asaedam@hiroshima-u.ac.jp)

To the Editor’s comments

In preparing your revised manuscript, please address the following key points in addition to the specific reviewer comments:

- **Clarify and refine your hypothesis and objectives** to more precisely reflect your focus on balance impairment (i.e., SLST) rather than general physical function.

**Response:** Thank you very much for your insightful comment. In response to your suggestion, we have revised the description of the hypothesis and objective to better clarify the focus of our study, particularly highlighting the single-leg stance test (SLST) as the primary outcome. The revised text in the manuscript is as follows:

**(Introduction, Page 5, Lines 88–91):**

"We hypothesized that comorbid DM is associated with impaired balance in patients with CLD and that this decline would be evident in the single‑leg stance test (SLST) as a measurement of balance. Therefore, the aim of this study was to examine whether comorbid DM is associated with impaired SLST in patients with CLD."

- **Revise and standardize methodological descriptions**, including the FIB-4 formula, consent procedure, variable definitions, and statistical approach (e.g., normality testing, multicollinearity diagnostics, use of exact logistic regression where appropriate).

**Response:** Thank you very much for your important and constructive comments. In response, we have clarified several methodological aspects, including the formula for calculating the FIB-4 index, the informed consent process, variable definitions, and our statistical approach (e.g., normality testing, multicollinearity diagnostics, and use of exact logistic regression when appropriate). These revisions have been made in multiple sections of the manuscript and we have included a copy below for your reference:

**(Methods, Page 5, Lines 103–112):**

"The study was conducted in accordance with Declaration of Helsinki and the Ethical Guidelines for Medical and Health Research Involving Human Subjects enacted by the Ministry of Health, Labour and Welfare of Japan (https://www.mhlw.go.jp/content/001077424.pdf), and approved by the Ethical Review Committee of Hiroshima University Hospital (Permit No. E-583-1). All patients were verbally informed that their medical records and charts might be used for research purposes. Data were obtained during routine medical care and reviewed retrospectively. The need to obtain informed consent from the study participants was waived by the Ethical Review Committee of Hiroshima University Hospital due to the retrospective nature of the study. Patients who were eligible for this study had the opportunity to refuse to participate in the study by opting out."

**(Methods, Page 7, Lines 134–138):**

“Liver fibrosis was assessed using serum marker-based scoring systems, including the AST-to-platelet ratio index (APRI) and the fibrosis-4 (FIB-4) index [49–53]. The APRI and FIB-4 index were calculated using the following equations,

$$\boldsymbol{APRI=}\frac{\boldsymbol{AST}\left[ \frac{\mathbf{I}\boldsymbol{U}}{\boldsymbol{L}} \right]\boldsymbol{/upper limit of normal AST}\left[ \frac{\mathbf{I}\boldsymbol{U}}{\boldsymbol{L}} \right]}{\boldsymbol{PLT}\left[ \frac{\boldsymbol{10}^{\boldsymbol{9}}}{\boldsymbol{L}} \right]}\boldsymbol{\times100}$$

$$\boldsymbol{FIB}\boldsymbol{-}\boldsymbol{4 index}\boldsymbol{=}\frac{\boldsymbol{AST}\left[ \frac{\boldsymbol{IU}}{\boldsymbol{L}} \right]\boldsymbol{\times age [years]}}{\boldsymbol{PLT}\left[ \frac{\boldsymbol{10}^{\boldsymbol{9}}}{\boldsymbol{L}} \right]\boldsymbol{\times}\sqrt{\boldsymbol{ALT}\left[ \frac{\boldsymbol{IU}}{\boldsymbol{L}} \right]}}$$

**(Methods, Page 10, Lines 204–217):**

“All continuous variables were first subjected to the Shapiro–Wilk test to assess normality. Variables that followed a normal distribution were presented as mean (± standard deviation) and were compared between groups using the Student’s t-test. Variables that deviated from normality were expressed as median (interquartile range) and compared using the Mann–Whitney U test. Categorical variables were presented as absolute numbers and percentages and compared using the Fisher’s exact test or chi-squared test, as appropriate.

An additional analysis was conducted using data from 118 patients with CLD who had comorbid DM, to identify factors associated with impaired SLST in this subgroup. Logistic regression analysis was performed with impaired SLST (< 5 s) as the dependent variable, using both univariate and multivariate models, and ORs with 95% CIs were reported. Following previous studies, KEF/weight values were multiplied by 100 and ECW/TBW values by 1000 to facilitate interpretation [66]. Multicollinearity was assessed by calculating variance inflation factors (VIFs), with values ≥ 2 considered indicative of collinearity.”

- **Expand your discussion** to contextualize the ECW/TBW findings. Clearly note its current status as a data-derived marker needing external validation, and consider integrating relevant mechanistic pathways.

**Response:** Thank you for these valuable suggestions. In response, we have expanded the discussion to better contextualize the significance of ECW/TBW. In particular, we have clarified that ECW/TBW is a data-derived marker that requires external validation, and we have incorporated relevant mechanistic pathways to support its interpretation. Specifically, we have made the following additions:

**(Discussion, Page 24, Lines 395–419):**

" The association between ECW/TBW and SLST in patients with CLD and comorbid DM may be explained by the interplay of multiple pathophysiological mechanisms. In liver cirrhosis, portal hypertension and lymphatic dysfunction lead to the accumulation of interstitial fluid [79, 80], resulting in pronounced lower extremity edema [81]. Such edema may impair muscle strength and proprioception, potentially leading to diminished balance capabilities [82-84]. Furthermore, CLD with comorbid DM is characterized by a significant resistance to insulin that has been linked to reductions in skeletal muscle mass and strength [85, 86]. In mice lacking insulin receptors (IR) and insulin-like growth factor-I receptors (IGF-IR) in skeletal muscles, protein catabolism is enhanced through activation of the proteasome and autophagy pathways, leading to muscle wasting and weakness. Notably, muscle atrophy is suppressed in mice deficient in Forkhead box O, indicating the critical role of IR/IGF-IR/Forkhead Box O signaling in maintaining muscle mass and strength [87]. In addition, mice lacking insulin signaling molecules such as phosphoinositide 3-kinase [88] and Akt [89] exhibit not only impaired glucose tolerance but also reductions in muscle mass and fiber diameter, further supporting the relationship between insulin resistance and muscle dysfunction. Myostatin, a myokine that strongly suppresses muscle protein synthesis, plays a key role in maintaining muscle homeostasis in accordance with anabolic myokines [90]. In patients with CLD, serum myostatin levels tend to increase as the disease progresses, potentially contributing to suppressed muscle protein synthesis [91, 92]. Taken together, these findings suggest that changes in interstitial fluid dynamics, insulin resistance-induced impairment of muscle signaling, and disruption of liver–muscle crosstalk act in concert to link increased ECW/TBW with impaired SLST. The relationship between decreased SLST and elevated ECW/TBW demonstrated in this study is not a mere correlation, but rather reflects a comprehensive pathophysiological process involving fluid imbalance, disrupted metabolic signaling, and changes in muscle quality."

**(Discussion, Page 26, Lines 430–433):**

“Finally, the ECW/TBW cutoff value of 0.393 was derived from the present dataset, and external validation is needed prior to clinical application. Future prospective studies should aim to collect more detailed data on these variables to confirm and expand upon our findings.”

- **Ensure consistency** in terminology (e.g., always use “diabetes mellitus (DM)” after first mention), abbreviation use, and statistical formatting (e.g., p-values).

**Response:** Thank you for your helpful comment. We have revised the manuscript to ensure consistency in terminology. Specifically, we used the full term “diabetes mellitus (DM)” at its first appearance and used the abbreviation “DM” thereafter throughout the text. Additionally, we standardized the presentation of *p*-values to either “*p* = 0.xxx” or “*p* < 0.001” as appropriate.

To the Journal requirements

1. Please ensure that your manuscript meets PLOS ONE's style requirements, including those for file naming. The PLOS ONE style templates can be found at [https://journals.plos.org/plosone/s/file?id=wjVg/PLOSOne_formatting_sample_main_body.pdf](https://track.editorialmanager.com/CL0/https:%2F%2Fjournals.plos.org%2Fplosone%2Fs%2Ffile%3Fid=wjVg%2FPLOSOne_formatting_sample_main_body.pdf/1/010f019658c9704c-391cc9d2-f81e-4db5-b7df-7265b10681ad-000000/K6qxKy7HnsLRSEJgzwPuqRqjqEMRzF4zudRQiAYJqyA=207)  and [https://journals.plos.org/plosone/s/file?id=ba62/PLOSOne_formatting_sample_title_authors_affiliations.pdf](https://track.editorialmanager.com/CL0/https:%2F%2Fjournals.plos.org%2Fplosone%2Fs%2Ffile%3Fid=ba62%2FPLOSOne_formatting_sample_title_authors_affiliations.pdf/1/010f019658c9704c-391cc9d2-f81e-4db5-b7df-7265b10681ad-000000/rkDfguSI35wGtIq-bH53B6QZDJ6OOIi-rBunXff1gP8=207)

**Response:** Thank you for your valuable comment. We have adjusted the manuscript formatting, including file naming, in accordance with the PLOS ONE style requirements.

2. Thank you for stating the following financial disclosure:

“This work was supported by a grant (H30-A59) from the Japanese Society of Physical Therapy and Japan Society for the Promotion of Science KAKENHI Grant Number JP19K19831.”

Please state what role the funders took in the study.  If the funders had no role, please state: "The funders had no role in study design, data collection and analysis, decision to publish, or preparation of the manuscript."

If this statement is not correct you must amend it as needed.

Please include this amended Role of Funder statement in your cover letter; we will change the online submission form on your behalf.

**Response:** Thank you for your confirmation. We have included the following statement regarding the role of the funders in the cover letter:

**(Response letter, Page 1)**

"The funders had no role in study design, data collection and analysis, decision to publish, or preparation of the manuscript."

3. In this instance it seems there may be acceptable restrictions in place that prevent the public sharing of your minimal data. However, in line with our goal of ensuring long-term data availability to all interested researchers, PLOS’ Data Policy states that authors cannot be the sole named individuals responsible for ensuring data access (<http://journals.plos.org/plosone/s/data-availability#loc-acceptable-data-sharing-methods>).

Data requests to a non-author institutional point of contact, such as a data access or ethics committee, helps guarantee long term stability and availability of data. Providing interested researchers with a durable point of contact ensures data will be accessible even if an author changes email addresses, institutions, or becomes unavailable to answer requests.

Before we proceed with your manuscript, please also provide non-author contact information (phone/email/hyperlink) for a data access committee, ethics committee, or other institutional body to which data requests may be sent. If no institutional body is available to respond to requests for your minimal data, please consider if there any institutional representatives who did not collaborate in the study, and are not listed as authors on the manuscript, who would be able to hold the data and respond to external requests for data access? If so, please provide their contact information (i.e., email address). Please also provide details on how you will ensure persistent or long-term data storage and availability.

**Response:** Thank you for your comment. We confirm that data access will not be solely managed by the authors. We have arranged with the Hiroshima University Library to archive the dataset in the Hiroshima University Institutional Repository (https://ir.lib.hiroshima-u.ac.jp), and they have agreed to act as the non-author institutional contact for data access inquiries. Please direct any inquiries regarding the dataset to:

**Hiroshima University Library**

**Email: tosho-soumu@office.hiroshima-u.ac.jp**

**Website:** [**https://www.lib.hiroshima-u.ac.jp/**](https://www.lib.hiroshima-u.ac.jp/)

The library has committed to long-term preservation and public access to the dataset, ensuring that data remain available even if authors become unreachable.

4. When completing the data availability statement of the submission form, you indicated that you will make your data available on acceptance. We strongly recommend all authors decide on a data sharing plan before acceptance, as the process can be lengthy and hold up publication timelines. Please note that, though access restrictions are acceptable now, your entire data will need to be made freely accessible if your manuscript is accepted for publication. This policy applies to all data except where public deposition would breach compliance with the protocol approved by your research ethics board. If you are unable to adhere to our open data policy, please kindly revise your statement to explain your reasoning and we will seek the editor's input on an exemption. Please be assured that, once you have provided your new statement, the assessment of your exemption will not hold up the peer review process.

**Response:** We confirm that the minimal dataset will be deposited in the Hiroshima University Institutional Repository and made freely and publicly accessible upon acceptance of the manuscript. The Hiroshima University Library has already agreed to this and the registration process is underway. We will update the Data Availability Statement in the manuscript as follows:

**Data Availability:**

“All relevant data will be made available without restriction in the Hiroshima University Institutional Repository (https://ir.lib.hiroshima-u.ac.jp) following acceptance. Data requests may also be directed to Hiroshima University Library (tosho-soumu@office.hiroshima-u.ac.jp), which will manage long-term data access.”

5. Please amend either the title on the online submission form (via Edit Submission) or the title in the manuscript so that they are identical.

**Response:** Thank you for your valuable comment. We have standardized the manuscript title in both the online submission system and within the manuscript itself as follows:

**(Page 1, Lines 4–5)**

*“Association between diabetes mellitus and impaired single-leg stance in patients with chronic liver disease: a cross-sectional study”*

To the Reviewer: 1

Introduction (Lines 48–72)

- Line 67: Reference specific studies showing increased fall risk due to diabetic neuropathy or proprioceptive impairment.

**Response:** Thank you very much for your valuable comment. In accordance with your suggestion, we have added several key references to support this point. The revised manuscript now states:

**(Introduction, Page 4, Lines 82–86):**

"Patients with DM also experience impaired balance due to factors such as peripheral neuropathy, hypoglycemia, and delayed reaction times [42-44]; the presence of DM alone increases the risk of falls [45]. Peripheral neuropathy and proprioceptive impairment have been identified as independent risk factors for falls [46-48]. Notably, neuropathy associated with DM has also been shown to elevate the risk of falls in affected patients [42]."

- Line 71: Replace “poorer physical function” with “reduced physical function” for clarity and academic tone.

**Response:** We appreciate the opportunity to clarify phrasing in the manuscript. To enhance clarity and academic tone, we have replaced the phrase “poorer physical function” with more precise terms such as “declines in physical function” and “impaired physical function” throughout the manuscript. In the revised version, the relevant section now includes:

**(Introduction, Page 5, Lines 71–82):**

"As CLD progresses, the occurrence of sarcopenia increases. Several reports have documented declines in physical function, such as grip strength and gait speed, in patients with advanced CLD [3, 28–33]. CLD, particularly cirrhosis, is associated with an increased risk of falls. Notably, even in the absence of hepatic encephalopathy, the annual fall incidence has been reported as 27.2% in patients under 60 years of age and 30.5% in those aged 60 and above [34]. These rates substantially exceed those observed in large-scale community-based populations, which are 11.4% among individuals aged 45–64 years and 16.4% among those aged 65 years or older [35]. Cirrhosis is also associated with increased healthcare costs and greater caregiver burden [36]. DM further exacerbates these issues by increasing the prevalence of sarcopenia and impairing physical function [37, 38]. Moreover, DM-related complications, such as nephropathy [39] and neuropathy [40, 41], can further contribute to compromised physical function.”

- Line 72: The objective could be rephrased more precisely: “to examine whether comorbid diabetes mellitus is associated with impaired balance, as measured by single-leg standing time, in patients with chronic liver disease.”

**Response:** Thank you very much for your valuable suggestion. As also pointed out in Editor’s Comment 1, we have revised the description of the study objective. Additionally, to improve consistency, we unified the terminology by replacing the previously mixed usage of “balance ability” and “balance function” with “balance ability” throughout the manuscript. In the revised version, the relevant section of text now reads as follows:

**(Introduction, Page 5, Lines 87–91):**

“Despite the high prevalence of DM among patients with CLD, its impact on physical function remains unclear. We hypothesized that comorbid DM is associated with impaired balance in patients with CLD and that this decline would be evident in the single‑leg stance test (SLST) as a measurement of balance. Therefore, the aim of this study was to examine whether comorbid DM is associated with impaired SLST in patients with CLD.”

Methods (Lines 74–192)
Line 84: Provide justification for the opt-out consent model. Mention whether this aligns with national research guidelines for retrospective studies in Japan.

**Response:** Thank you very much for your insightful comment. We have added an explanation regarding the validity of the opt-out consent model, including its alignment with Japanese research guidelines. The revised text is:

**(Methods, Page 5, Lines 103–112):**

“The study was conducted in accordance with Declaration of Helsinki and the Ethical Guidelines for Medical and Health Research Involving Human Subjects enacted by the Ministry of Health, Labour and Welfare of Japan (https://www.mhlw.go.jp/content/001077424.pdf), and approved by the Ethical Review Committee of Hiroshima University Hospital (Permit No. E-583-1). All patients were verbally informed that their medical records and charts might be used for research purposes. Data were obtained during routine medical care and reviewed retrospectively. The need to obtain informed consent from the study participants was waived by the Ethical Review Committee of Hiroshima University Hospital due to the retrospective nature of the study. Patients who were eligible for this study had the opportunity to refuse to participate in the study by opting out.”

- Lines 96–98: There is repetition in the list of blood markers (e.g., AST, ALT, ALB) — revise to eliminate redundancy.

**Response:** Thank you for your valuable observation. We have removed the duplicated items (AST, ALT, ALB) and revised the list accordingly, as shown below:

**(Methods, Page 6, Lines 124–128):**
“Patient age, sex, height, weight, body mass index (BMI), blood data (hemoglobin [HGB], platelet [PLT] counts, total bilirubin [T-BIL], aspartate aminotransferase [AST], alanine aminotransferase [ALT], albumin [ALB], prothrombin time [PT], ammonia [NH₃], triglycerides [TG], uric acid [UA], and hemoglobin A1c) were extracted from medical records.”

- Line 109: The FIB-4 formula includes a square root of ALT, which is not conventional. Please clarify with a reference or correct if inaccurate.

**Response:** Thank you very much for your insightful comment. We acknowledge that our original description of the APRI and FIB-4 indices was unclear. In response, we have clearly presented the formulas for both indices in the revised manuscript. Additionally, we have cited the seminal work by Sterling et al. (2006), who originally proposed the FIB-4 index, to provide context and evidence for its use. The revised section reads as follows:

**(Methods, Page 7, Lines134–138):**

“Liver fibrosis was assessed using serum marker-based scoring systems, including the AST-to-platelet ratio index (APRI) and the fibrosis-4 (FIB-4) index [49–53]. The APRI and FIB-4 index were calculated using the following equations,

$$\boldsymbol{APRI=}\frac{\boldsymbol{AST}\left[ \frac{\boldsymbol{IU}}{\boldsymbol{L}} \right]\boldsymbol{/upper limit of normal AST}\left[ \frac{\boldsymbol{IU}}{\boldsymbol{L}} \right]}{\boldsymbol{PLT}\left[ \frac{\boldsymbol{10}^{\boldsymbol{9}}}{\boldsymbol{L}} \right]}\boldsymbol{\times100}$$

$$\boldsymbol{FIB-4 index=}\frac{\boldsymbol{AST}\left[ \frac{\boldsymbol{IU}}{\boldsymbol{L}} \right]\boldsymbol{\times age [years]}}{\boldsymbol{PLT}\left[ \frac{\boldsymbol{10}^{\boldsymbol{9}}}{\boldsymbol{L}} \right]\boldsymbol{\times}\sqrt{\boldsymbol{ALT}\left[ \frac{\boldsymbol{IU}}{\boldsymbol{L}} \right]}}$$

- Line 126: Specify whether SLST measurements had inter-rater or intra-rater reliability assessments.

**Response**: Thank you very much for your valuable suggestion to improve the manuscript. We have added a clarification regarding the reliability of the single-leg stance test (SLST) measurement in the revised manuscript. Specifically, we now state the following:

**(Methods, Page 8, Lines 154–160):**

"The SLST is recognized as a reliable method with high inter-rater reliability, particularly when the maximum value from multiple trials is used [55]. Previous studies have reported no significant difference in balance between the dominant and non-dominant legs during the SLST [56]. As our participants did not have any musculoskeletal or neuromuscular disorders, we used the average of the maximum values from each leg for the analysis. To account for potential concerns regarding side-to-side differences, we also calculated the maximum value for each leg individually."

- Lines 170–173: Confirm whether normality of distribution was assessed before choosing statistical tests like t-tests or Mann-Whitney U. If so, report those results explicitly.

**Response:** Thank you for your insightful comment. In response, we have revised the Methods section to clarify that all continuous variables were tested for normality using the Shapiro-Wilk test prior to selecting appropriate statistical procedures. Specifically, we added the following sentence:

**(Methods, Page 10, Lines 204–209):**

“All continuous variables were first subjected to the Shapiro–Wilk test to assess normality. Variables that followed a normal distribution were presented as mean (± standard deviation) and were compared between groups using the Student’s t-test. Variables that deviated from normality were expressed as median (interquartile range) and compared using the Mann–Whitney U test. Categorical variables are presented as absolute numbers and percentages, and were compared using the Fisher’s exact test or chi-squared test, as appropriate.”

Results (Lines 194–295)

- Line 199: Consider merging etiologies with low sample size (e.g., HBV, ALD, others) or acknowledging the limited power for these subgroups.

**Response:** Thank you for your valuable comment. We recognize the limitations in statistical power associated with detecting rare etiologies in our sample. In response, we have added this point to the limitations section of the manuscript as follows:

**(Discussion, Page 26, Lines 426–428):**

"Fourth, the small sample sizes in the HBV, ALD, and other etiology groups limited our ability to detect statistically significant differences between subgroups "

- Line 243: A 0% vs. 20% SLST impairment rate is striking — discuss whether key confounders (e.g., diabetic neuropathy, medication use, or fall history) were considered or controlled.

**Response:** Thank you for your insightful comment. In response, we have acknowledged the limitations regarding the lack of data collection on potentially important confounding factors and have added this to the limitations section of the revised manuscript. Specifically, we included the following sentence:

**(Discussion, Page 26, Lines 424–426):**

"Third, detailed information regarding the severity of DM was lacking, preventing us from evaluating the potential influence of DM duration, diabetic neuropathy, medication use, and fall history. "

- Line 282: The predictive value of ECW/TBW (AUC = 0.733, PPV = 37%) is moderate. Discuss whether combining this marker with other functional metrics (e.g., grip strength) could improve diagnostic accuracy.

**Response:** Thank you very much for your valuable suggestion. We have emphasized the usefulness of ECW/TBW as an exclusion marker and referred to the combination with lower limb muscle strength as follows:

**(Discussion, Page 23, Lines 372–394):**

“Given that lower extremity muscle strength is more closely associated with walking ability than grip strength [74], we conducted ROC analysis on a multivariable model incorporating age, BMI, ECW/TBW, and KEF/weight in relation to impaired SLST. Although the four-variable model achieved an AUC of 0.795, ECW/TBW alone also demonstrated acceptable predictive performance. Therefore, considering its high NPV (91.4%), an ECW/TBW cutoff of 0.393 appears well suited as an exclusion marker for impaired SLST. ECW/TBW increases as liver fibrosis progresses and has been reported as a marker of liver dysfunction and prognosis in patients with cirrhosis [75]. It has also been suggested that ECW/TBW gradually increases in the pre-cirrhotic stage [75]. A threshold of ECW/TBW ≥ 0.400 is considered indicative of a moderate-to-severe overhydrated state [76]. Several studies have reported that edema negatively affects physical function, including gait ability [77, 78]. Similarly, in our study, increased ECW/TBW was associated with impaired SLST, suggesting that lower limb edema may contribute to reduced balance. Furthermore, ECW/TBW of 0.393 was identified as a potential predictor for ruling out impaired SLST in patients with CLD and comorbid DM. Previous studies have suggested that DM is associated with fluid retention in patients with CLD [14, 24]. Notably, DM has been shown to be independently associated with ascites development in cirrhotic patients, irrespective of the model used for end-stage liver disease scores [24]. The underlying mechanisms of DM-associated fluid retention in CLD remain unclear, however, alterations in hepatic and renal microcirculation have been implicated [14]. From a rehabilitation perspective, even in cases where ECW/TBW does not exceed 0.400, values of 0.390–0.400 may represent a “subclinical” fluid retention state. It may be necessary to reconsider mild overhydration as an important factor in ruling out the risk of falls in patients with CLD and comorbid DM.”

Discussion (Lines 297–366)

- Line 304: Reaffirm how this is the first study (to your knowledge) evaluating SLST in CLD populations — cite any comparable work or clarify novelty.

**Response:** Thank you kindly for this positive feedback and suggestion. We have clarified the novelty of the present study as follows:

**(Discussion, Page 22, Lines 340–341):**

“To the best of our knowledge, this is the first study to evaluate SLST as an indicator of balance in this patient population.”

- Line 330: Address the low variance in ammonia levels (NH₃), which may explain the lack of association with SLST. Suggest this as a sample bias or a limitation of the study cohort.

**Response:** Thank you very much for your valuable comment. We have added a description in the limitations section regarding the limited variability of NH₃ levels, as follows:

**(Discussion, Page 26, Lines 428–429):**

“Fifth, the limited variability in NH₃ levels among our participants, reflecting predominantly mild CLD, may have obscured any association with SLST.”

- Line 337: Include references to prior research connecting mild overhydration or edema to proprioceptive or postural instability to support the ECW/TBW mechanism.

**Response:** Thank you very much for your valuable suggestion. To further contextualize the association between ECW/TBW and balance ability, we conducted a more comprehensive review of the literature and expanded the discussion accordingly, as follows:

**(Introduction, Page 4, Lines 82–86)**

“Patients with DM also experience impaired balance due to factors such as peripheral neuropathy, hypoglycemia, and delayed reaction times [42–44]; the presence of DM alone increases the risk of falls [45]. Peripheral neuropathy and proprioceptive impairment have been identified as independent risk factors for falls [46–48]. Notably, neuropathy associated with DM has been shown to elevate the risk of falls in affected patients [42].”

**(Discussion, Page 24, Lines 395–419):**

“

The association between ECW/TBW and SLST in patients with CLD and comorbid DM may be explained by the interplay of multiple pathophysiological mechanisms. In liver cirrhosis, portal hypertension and lymphatic dysfunction lead to the accumulation of interstitial fluid [79, 80], resulting in pronounced lower extremity edema [81]. Such edema may impair muscle strength and proprioception, potentially leading to diminished balance capabilities [82-84]. Furthermore, CLD with comorbid DM is characterized by a significant resistance to insulin that has been linked to reductions in skeletal muscle mass and strength [85, 86]. In mice lacking insulin receptors (IR) and insulin-like growth factor-I receptors (IGF-IR) in skeletal muscles, protein catabolism is enhanced through activation of the proteasome and autophagy pathways, leading to muscle wasting and weakness. Notably, muscle atrophy is suppressed in mice deficient in Forkhead box O, indicating the critical role of IR/IGF-IR/Forkhead Box O signaling in maintaining muscle mass and strength [87]. In addition, mice lacking insulin signaling molecules such as phosphoinositide 3-kinase [88] and Akt [89] exhibit not only impaired glucose tolerance but also reductions in muscle mass and fiber diameter, further supporting the relationship between insulin resistance and muscle dysfunction. Myostatin, a myokine that strongly suppresses muscle protein synthesis, plays a key role in maintaining muscle homeostasis in accordance with anabolic myokines [90]. In patients with CLD, serum myostatin levels tend to increase as the disease progresses, potentially contributing to suppressed muscle protein synthesis [91, 92]. Taken together, these findings suggest that changes in interstitial fluid dynamics, insulin resistance-induced impairment of muscle signaling, and disruption of liver–muscle crosstalk act in concert to link increased ECW/TBW with impaired SLST. The relationship between decreased SLST and elevated ECW/TBW demonstrated in this study is not a mere correlation, but rather reflects a comprehensive pathophysiological process involving fluid imbalance, disrupted metabolic signaling, and changes in muscle quality.

”

- Line 349: Recommend specific design elements for future research, e.g., prospective follow-up with diabetes duration, neuropathy scores, and functional falls data.

**Response:** Thank you very much for your valuable suggestion. We have incorporated your recommendation regarding future study design into the limitations section of the manuscript, as follows:

**(Discussion, Page 26, Lines 4248–433)**

“Third, detailed information regarding the severity of DM was unavailable, preventing us from evaluating the potential influence of DM duration, diabetic neuropathy, medication use, and fall history. Fourth, the small sample sizes in the HBV, ALD, and other etiology groups limited our ability to detect significant differences between subgroups. Fifth, the limited variability in NH₃ levels among our participants, reflecting predominantly mild CLD, may have obscured any association with SLST. Therefore, the possibility of selection bias cannot be excluded. Sixth, the ECW/TBW cutoff value of 0.393 was derived from the present dataset, and external validation is needed prior to clinical application. Future prospective studies should aim to collect more detailed data on these variables to validate and expand upon our findings.”

General Suggestions

- Consistency: Standardize all uses of “diabetes” to “diabetes mellitus (DM)” after first mention for clarity.

**Response:** Thank you for your insightful comment. In response, we have standardized the terminology throughout the manuscript by replacing all instances of “diabetes” with “diabetes mellitus.” After spelling it out at the first occurrence, we consistently used the abbreviation “DM” thereafter, in accordance with academic conventions.

- Abbreviations: Suggest adding a footnote or separate table listing abbreviations like ECW/TBW, ASM/BMI, KEF, etc.

**Response:** Thank you for your valuable suggestion. In response, we carefully reviewed all abbreviations used in the main text, figures, and tables. We have revised the footnotes to ensure consistency and reader understanding throughout the manuscript.

- Statistics: Ensure consistent p-value formatting (e.g., p = 0.002). If exact p-values are unavailable, use p < 0.001 rather than zero.

**Response:** Thank you for your comment, and we apologize for any confusion in p-value formatting in the original manuscript. In accordance with your suggestion, we have standardized the presentation of p-values throughout the manuscript. Specifically, we now consistently use the format “p = 0.xxx” or “p < 0.001” as appropriate.

To the Reviewer: 2

Introduction:

- The hypothesis is stated as a general decline in “physical function” among DM patients with CLD. However, the actual analysis focuses narrowly on balance function as assessed by SLST. I suggest reframing the hypothesis to specifically address postural stability or balance-related impairment, aligning better with the operationalized outcome.

**Response:** Thank you very much for your insightful comment. As you correctly pointed out, there was a discrepancy between the stated hypothesis and the actual analytical focus of the study. To address this issue, we have revised the Introduction section to clarify and specify our hypothesis, explicitly focusing on balance ability as assessed by the single-leg stance test (SLST). The revised text is as follows:

**(Introduction, Page 5, Lines 87–91):**

“Despite the high prevalence of DM among patients with CLD, its impact on physical function remains unclear. We hypothesized that comorbid DM is associated with impaired balance in patients with CLD and that this decline would be evident in the single‑leg stance test (SLST) as a measurement of balance. Therefore, the aim of this study was to examine whether comorbid DM is associated with impaired SLST in patients with CLD.”

Methods:

- The FIB-4 formula includes a square root of ALT, which deviates from the canonical form (without transformation). This must be clarified, as it could affect the fibrosis score interpretation across studies.

**Response:** Thank you very much for your valuable comment. We apologize for the lack of clarity regarding the descriptions of the APRI and FIB-4 index in the manuscript. In response, we have revised and standardized the format of these variables to improve readability. Additionally, we have cited the seminal paper by Sterling et al. (2006), which originally proposed the FIB-4 index. We hope this revision clarifies the methodology and enhances the scientific rigor of our manuscript.

**(Methods, Page 7, Lines 134–138):**

“

Liver fibrosis was assessed using serum marker-based scoring systems, including the AST-to-platelet ratio index (APRI) and the fibrosis-4 (FIB-4) index [49–53]. The APRI and FIB-4 index were calculated using the following equations,

$$\boldsymbol{APRI=}\frac{\boldsymbol{AST}\left[ \frac{\boldsymbol{IU}}{\boldsymbol{L}} \right]\boldsymbol{/upper limit of normal AST}\left[ \frac{\boldsymbol{IU}}{\boldsymbol{L}} \right]}{\boldsymbol{PLT}\left[ \frac{\boldsymbol{10}^{\boldsymbol{9}}}{\boldsymbol{L}} \right]}\boldsymbol{\times100}$$

$$\boldsymbol{FIB-4 index=}\frac{\boldsymbol{AST}\left[ \frac{\boldsymbol{IU}}{\boldsymbol{L}} \right]\boldsymbol{\times age [years]}}{\boldsymbol{PLT}\left[ \frac{\boldsymbol{10}^{\boldsymbol{9}}}{\boldsymbol{L}} \right]\boldsymbol{\times}\sqrt{\boldsymbol{ALT}\left[ \frac{\boldsymbol{IU}}{\boldsymbol{L}} \right]}}$$

“

- SLST methodology uses the mean of both legs, but no indication is given about distributional skew or floor effects. Given that 20% of DM patients had SLST <5 seconds and non-DM had 0%, consider whether a dichotomous transformation (e.g., <5s vs ≥5s) might yield more interpretable or clinically meaningful stratification for this population.

**Response:** Thank you very much for your insightful comment. As you correctly pointed out, applying a multivariate analysis across both groups would be inappropriate given the zero-cell count in the non-DM group. Therefore, we conducted the analysis only within the DM group to identify factors associated with impaired balance. To avoid confusion, we have now clearly stated in the manuscript that this is a subgroup analysis limited to patients with DM.

**(Materials and Methods, Page 10, Lines 210–217)**

“An additional analysis was conducted using data from 118 patients with CLD who had comorbid DM, to identify factors associated with impaired SLST in this subgroup. Logistic regression analysis was performed with impaired SLST (< 5 s) as the dependent variable, using both univariate and multivariate models, and ORs with 95% CIs were reported. Following previous studies, KEF/weight values were multiplied by 100 and ECW/TBW values by 1000 to facilitate interpretation [65]. Multicollinearity was assessed by calculating variance inflation factors (VIFs), with values ≥ 2 considered indicative of collinearity.”

- It is unclear whether leg dominance was recorded. While cited literature claims no performance difference, individual patient-level variability may still affect balance metrics, particularly in neuromuscular-compromised cohorts.

**Response:** Thank you very much for your thoughtful comment. Chronic liver disease (CLD) is not classified as a neuromuscular or musculoskeletal disorder, and in our study, serum ammonia (NH₃) levels were low with limited variability among participants. Therefore, we believe that concerns related to neuromuscular impairment are minimal in this cohort. Nevertheless, to address your point and enhance data transparency, we additionally calculated the maximum value between the left and right limbs and included this information in Tables 1 and 3.

**(Materials and Methods, Page 8, Lines 147–153), Tables 1, 3**

“The SLST is recognized as a reliable method with high inter-rater reliability, particularly when the maximum value from multiple trials is used [57]. Previous studies have reported no significant difference in balance between the dominant and non-dominant legs during the SLST [58]. As our participants did not have any musculoskeletal or neuromuscular disorders, we used the average of the maximum values from each leg for the analysis. To account for potential concerns regarding lateral differences, we also calculated the maximum value for each leg individually.”

- There is no mention of multicollinearity diagnostics before entering variables into multivariate logistic regression. BMI and ECW/TBW may be collinear. Reporting variance inflation factors (VIFs) or tolerance values is recommended.

**Response:** Thank you very much for your valuable comment. We acknowledge that our initial manuscript lacked a clear explanation regarding the assessment of multicollinearity. In response to your suggestion, we have now included the evaluation of multicollinearity using variance inflation factors (VIFs). The relevant description has been added to the Methods section, and the VIF values for all explanatory variables included in the logistic regression model were confirmed to be below 5, indicating no significant multicollinearity.

**(Materials and Methods, Page 11, Lines 216–217):**

"Multicollinearity was assessed by calculating variance inflation factors (VIFs), with values ≥2 considered indicative of collinearity.

**(Results, Page 19, Lines 297–298), Table 4:**

“Furthermore, the VIF values did not exceed 2 for any factor, suggesting the absence of multicollinearity.”

Results:

- The finding of 0% SLST <5s in non-DM patients vs. 20% in DM patients is striking but statistically vulnerable due to zero-cell counts. Consider whether exact logistic regression would be more appropriate than asymptotic methods here.

**Response:** Thank you very much for your insightful comment regarding the potential statistical issue related to the presence of a zero-cell in the cross-tabulation of SLST <5 s between patients with and without diabetes mellitus (DM). We appreciate your concern.

We would like to clarify that this cross-tabulation was used solely for descriptive purposes to illustrate the prevalence of impaired SLST in each group. While it is true that there were no cases of SLST <5 s in the non-DM group, our primary analysis focused exclusively on patients with chronic liver disease (CLD) complicated by DM. Within this subgroup, we performed both univariate and multivariate logistic regression analyses with SLST <5 s as the dependent variable, using continuous predictors such as ECW/TBW and BMI. These models were constructed without incorporating any data from the non-DM group.

Therefore, the issue of a zero-cell in the descriptive comparison does not affect the main logistic regression analysis, and there is no concern regarding biased maximum likelihood estimates. As a result, we believe that the use of exact logistic regression is not required in this context, and the application of conventional logistic regression and ROC analysis remains valid.

Nevertheless, we acknowledge that the subgroup nature of our analysis was not clearly described in the initial manuscript. We have now revised the Methods section to explicitly state that logistic regression and ROC analysis were conducted solely within the DM group, as follows:

**(Materials and Methods, Page 10, Lines 6210–217):**

“An additional analysis was conducted using data from 118 patients with CLD who had comorbid DM, to identify factors associated with impaired SLST in this subgroup. Logistic regression analysis was performed with impaired SLST (< 5 s) as the dependent variable, using both univariate and multivariate models, and ORs with 95% CIs were reported. Following previous studies, KEF/weight values were multiplied by 100 and ECW/TBW values by 1000 to facilitate interpretation [66]. Multicollinearity was assessed by calculating variance inflation factors, with values ≥ 2 considered indicative of collinearity.”

- The AUC for ECW/TBW is 0.733 with relatively low PPV (37%) but high NPV (91.4%). This indicates that ECW/TBW may serve better as a rule-out marker than a rule-in diagnostic. The authors should address this explicitly in interpretation.

**Response:** Thank you very much for your valuable comment. As you pointed out, although ECW/TBW had a moderate AUC of 0.733 and a relatively low positive predictive value (PPV) of 37%, it showed a high negative predictive value (NPV) of 91.4%. In response, we have explicitly revised the manuscript to highlight the potential utility of ECW/TBW as an **exclusion marker** for impaired single-leg stance.

Specifically, we have included the following statements to the manuscript:

**(Discussion, Page 23, Lines 372–377):**

“Given that lower extremity muscle strength is more closely associated with walking ability than grip strength [74], we conducted ROC analysis on a multivariable model incorporating age, BMI, ECW/TBW, and KEF/weight in relation to impaired SLST. Although the four-variable model achieved an AUC of 0.795, ECW/TBW alone also demonstrated acceptable predictive performance. Therefore, considering its high NPV (91.4%), an ECW/TBW cutoff of 0.393 appears well suited as an exclusion marker for impaired SLST.”

**(Discussion, Page 24, Lines 392–394):**

“It may be necessary to reconsider mild overhydration as an important factor in ruling out the risk of falls in patients with CLD and comorbid DM.”

**(Discussion, Page 26, Lines 430–433)**

“Finally, the ECW/TBW cutoff value of 0.393 was derived from the present dataset, and external validation is needed prior to clinical application.”

**(Conclusions, Page 26, Lines 441–443)**

“From a fall-prevention perspective, it may be necessary to reconsider mild overhydration (ECW/TBW 0.39–0.40) as an important factor in ruling out the risk of falls in patients with CLD and comorbid DM.”

Discussion (Lines 297–366)

- The discussion of ECW/TBW as "subclinical edema" is compelling. However, the threshold of 0.393 is data-derived from this study and requires external validation before being used in clinical settings. This caveat should be emphasized.

**Response:** Thank you very much for your feedback and suggestion for improvement to the manuscript. As you correctly pointed out, the ECW/TBW threshold of 0.393 was derived from our dataset and therefore requires external validation before it can be applied in clinical settings. We have revised the manuscript to clearly emphasize this limitation and the need for further validation.

Specifically, we have added the following sentence:

**(Discussion, Page 26, Lines 429–432)**

“Therefore, the possibility of selection bias cannot be excluded. Finally, the ECW/TBW cutoff value of 0.393 was derived from the present dataset, and external validation is needed prior to clinical application.”

- There’s an opportunity here to draw connections between interstitial fluid dynamics, insulin resistance, and muscle signaling pathways, all of which are altered in CLD-DM. These links could provide a more mechanistic narrative for the ECW/TBW-SLST association.

**Response:** Thank you very much for your valuable suggestion. In response, we have expanded the Discussion section to provide a more detailed, mechanism-based explanation of the interrelationships among interstitial fluid dynamics, insulin resistance, and muscle signaling pathways in patients with chronic liver disease complicated by diabetes mellitus (CLD-DM).

Specifically, we have added the following content:

**(Discussion, Page 24, Lines 395–419):**

“The association between ECW/TBW and SLST in patients with CLD and comorbid DM may be explained by the interplay of multiple pathophysiological mechanisms. In liver cirrhosis, portal hypertension and lymphatic dysfunction lead to the accumulation of interstitial fluid [79, 80], resulting in pronounced lower extremity edema [81]. Such edema may impair muscle strength and proprioception, potentially leading to diminished balance capabilities [82-84]. Furthermore, CLD with comorbid DM is characterized by a significant resistance to insulin that has been linked to reductions in skeletal muscle mass and strength [85, 86]. In mice lacking insulin receptors (IR) and insulin-like growth factor-I receptors (IGF-IR) in skeletal muscles, protein catabolism is enhanced through activation of the proteasome and autophagy pathways, leading to muscle wasting and weakness. Notably, muscle atrophy is suppressed in mice deficient in Forkhead box O, indicating the critical role of IR/IGF-IR/Forkhead Box O signaling in maintaining muscle mass and strength [87]. In addition, mice lacking insulin signaling molecules such as phosphoinositide 3-kinase [88] and Akt [89] exhibit not only impaired glucose tolerance but also reductions in muscle mass and fiber diameter, further supporting the relationship between insulin resistance and muscle dysfunction. Myostatin, a myokine that strongly suppresses muscle protein synthesis, plays a key role in maintaining muscle homeostasis in accordance with anabolic myokines [90]. In patients with CLD, serum myostatin levels tend to increase as the disease progresses, potentially contributing to suppressed muscle protein synthesis [91, 92]. Taken together, these findings suggest that changes in interstitial fluid dynamics, insulin resistance-induced impairment of muscle signaling, and disruption of liver–muscle crosstalk act in concert to link increased ECW/TBW with impaired SLST. The relationship between decreased SLST and elevated ECW/TBW demonstrated in this study is not a mere correlation, but rather reflects a comprehensive pathophysiological process involving fluid imbalance, disrupted metabolic signaling, and changes in muscle quality.”
