# Supplementary material for: Association between diabetes mellitus and impaired single-leg stance in patients with chronic liver disease: A cross-sectional study
Source: PLoS One. 2026 Mar 24;21(3):e0345407. doi: 10.1371/journal.pone.0345407 (PMC13012516; doi:10.1371/journal.pone.0345407)
Supplement: Attachment — Submitted filename: Response_to_Reviewers.docx [file pone.0345407.s006.docx]

March 2, 2026

Dr. Rafael Oliveira

Academic Editor

*PLOS ONE*

Dear Dr. Oliveira,

Thank you for the further review of our manuscript, **Manuscript ID**: PONE-D-25-15349R2, **Title**: Association between diabetes mellitus and impaired single-leg stance in patients with chronic liver disease: a cross-sectional study. We appreciate your helpful comments, which have allowed us to refine our work even further. We have addressed all your suggestions in the revised manuscript. Our point-by-point responses are provided below.

Best regards,

Makoto Asaeda

Department of Rehabilitation Medicine, Hiroshima University Hospital

1-2-3 Kasumi, Minami-ku, Hiroshima 734-8551, Japan TEL: +81-82-257-5566

E-mail: [asaedam@hiroshima-u.ac.jp](mailto:asaedam@hiroshima-u.ac.jp)

To Academic Editor’s Comments

The authors improved their work, but there is still an issue that avoid my recommendation of acceptance. Thus, I'll require another round of revisions.

Specifically, please consider the following comments:

**-L146-148**, "To ensure data consistency, all measurements were conducted during the daytime and at least 2 hours after the last meal." Can you support this methodological choice with references, since this is not a standard procedure for body composition analysis? Moreover, were some procedures regarding food and water intake before the assessment?;

**Response:** Thank you for this insightful comment. We have revised the Methods section to clarify our protocol and its rationale. **While a strict fasting period of at least 8 hours is often required for high-precision research, a 2-hour post-prandial interval is used in clinical practice to balance feasibility and data reliability.** We acknowledge that post-prandial impedance fluctuations can peak within several hours; however, clinical evidence suggests that the impact on skeletal muscle mass measurement is minimal and does not compromise the overall findings. Furthermore, we have clarified that measurements were taken after voiding and that patients were instructed to avoid strenuous exercise and excessive fluid intake before the assessment.

**(Methods, Page 7, Lines 138-146):**

“To ensure data consistency, all measurements were conducted during the daytime, after voiding, and at least 2 hours after the last meal. While a strict fasting period of at least 8 hours is typically required for high-precision research, a 2-hour post-prandial interval is commonly adopted in clinical settings for practical feasibility [26] [27]. This approach is supported by evidence from previous studies showing that, although meal-induced impedance fluctuations can peak within several hours after food intake, these biological changes result in only minor clinical measurement errors for skeletal muscle mass [26]. Additionally, participants were instructed to avoid strenuous exercise and excessive fluid intake prior to the assessment.”

**(References):**

“26	Korzilius JW, Oppenheimer SE, de Roos NM, Wanten GJ, Zweers H. Having breakfast has no clinically relevant effect on bioelectrical impedance measurements in healthy adults. Nutr J. 2023;22(1):55. doi: 10.1186/s12937-023-00882-5, PMID 37904176.

27.	Shibata K, Adachi T, Kameshima M, Kito H, Tanaka C, Sano T, et al. Prognostic impact of segmental extracellular water to total body water ratio in cardiovascular surgery patients. Clin Nutr. 2025;51:81-9. doi: 10.1016/j.clnu.2025.06.004, PMID 40554262.”

**-L220-221**, for Cohen's D, R and V Cramer's, references to support the values of interpretations are needed. In addition, rather than specific values, authors should be present ranges for each interpretation, for better clarity;

**Response:** Thank you for this suggestion. We have revised the Statistical Analysis section to present the interpretation of effect sizes as ranges rather than specific values, and we have added the appropriate reference.

**(Methods, Page 10, Lines 215-217):**

“Effect sizes were interpreted using the following criteria: Cohen’s *d* (0.2–0.5 = small, 0.5–0.8 = medium, and ≥ 0.8 = large), *r*, and Cramer’s *V* (0.1–0.3 = small, 0.3–0.5 = medium, and ≥ 0.5 = large) [38].”

**(References):**

“38.	Cohen J. Statistical power analysis for the behavioral sciences. routledge; 2013. doi: 10.4324/9780203771587..”

**-L438-439**, in theses sentences there are two "sixth" limitations. The second time should be replaced by "seventh".;

**Response:** We apologize for the typographical error in the numbering. We have corrected the numbering of the limitations; the point regarding NH₃ levels is now labeled as the "seventh" limitation, and the subsequent sequence has been verified for consistency.

**(Discussion , Page 25, Lines 434)**

“Seventh, the limited variability in NH₃ levels among our participants, reflecting predominantly mild CLD, may have obscured any association with SLST results.”

-96 references are too much for an original manuscript. This number should be reduced and not exceed 60.;

**Response:** In accordance with your request, we have significantly condensed the reference list. We removed redundant citations and consolidated molecular mechanism discussions by citing key review articles rather than multiple primary studies. The total number of references is now exactly 58.

**(Introduction, Page 4, Lines 69-76)**

“CLD progression increases the occurrence of sarcopenia, with a notable decline in physical function such as grip strength and gait speed [4] [8] [9]. Cirrhosis is associated with a high annual incidence of falls (approximately 30%), significantly exceeding rates observed in community-dwelling populations [9] [10]. Cirrhosis is also associated with increased healthcare costs and greater caregiver burden [11]. DM further exacerbates these issues by increasing sarcopenia prevalence and impairing muscle strength [12] [13]. Moreover, DM-related complications, particularly peripheral neuropathy, contribute to impaired balance and an elevated risk of falls [14] [15] [16].”

**(Discussion , Page 24, Lines 407-418)**

“The association between increased ECW/TBW and impaired SLST in patients with CLD and comorbid DM likely involves multiple pathophysiological mechanisms. In liver cirrhosis, portal hypertension and lymphatic dysfunction lead to interstitial fluid accumulation and lower extremity edema [5] [52], which may impair muscle strength and proprioception, thereby diminishing balance [53, 54]. Furthermore, insulin resistance—a hallmark of both CLD and DM—may activate proteasome and autophagy pathways via impaired signaling, potentially leading to muscle wasting and weakness [55] [56]. Additionally, elevated serum myostatin levels in progressive CLD may suppress muscle protein synthesis [57] [58]. Collectively, although the exact pathways remain unclear, changes in interstitial fluid dynamics, insulin resistance-induced signaling impairment, and disrupted liver–muscle crosstalk are potential factors linking increased ECW/TBW with impaired SLST..”

February 3, 2026

Dr. Rafael Oliveira

Academic Editor

*PLOS ONE*

Dear Dr. Sawaf,

Thank you for your consideration of our revised manuscript. We are grateful to the reviewers for their insightful and constructive feedback. We have addressed all the concerns raised by the editor and Reviewer #3. A point-by-point response is provided below. In this revised manuscript, we have addressed all the reviewers' concerns, specifically regarding the standardization of body composition assessment protocols, the clarification of statistical effect sizes, and the refinement of the study’s limitations. All changes in the revised manuscript are highlighted in red font.

Please ensure this correction is reflected in the author list and manuscript metadata upon acceptance.

We greatly appreciate the opportunity to revise our work and look forward to your response.

Best regards,

Makoto Asaeda

Department of Rehabilitation Medicine, Hiroshima University Hospital

1-2-3 Kasumi, Minami-ku, Hiroshima 734-8551, Japan TEL: +81-82-257-5566

E-mail: [asaedam@hiroshima-u.ac.jp](mailto:asaedam@hiroshima-u.ac.jp)

To Academic Editor’s Comments

The authors improved their by following the comments made by reviewer in the first division. However, there are still margin for improvements.

Therefore, another round of revisions will be requested. Please considerer the comments made by reviewers and see the following specific comments for more details:

**-in abstract,** please add effect sizes when it's appropriate;

**Response:** Thank you. We have added effect sizes to the Abstract.

**(Abstract, Page 3, Lines 44-48):**

“prevalence of impaired SLST than those without DM (20% vs. 0%, p = 0.002, Cramer’s *V* 0.23). In 118 patients with CLD who had comorbid DM, age (odds ratio [OR] 1.089, 95% confidence interval [CI] 1.020–1.177, p = 0.009), body mass index (OR 1.176, 95% CI 1.045–1.343, p = 0.006), and extracellular water-to-total body water ratio (ECW/TBW) (OR 1.065, 95% CI 1.003–1.138, p = 0.039) were significant independent factors associated with impaired SLST (Nagelkerke pseudo-*R²* 0.31, p < 0.001).”

**-L60-63** require at least one more citation or please rewrite the sentence;

**Response:** We have added the necessary citations to support the statements in the Introduction.

**(Introduction, Page 4, Lines 58–59)**

“Recent studies have highlighted the high prevalence of diabetes mellitus (DM) among patients with CLD [2, 3].”

**(References)**

“2.	Elkrief L, Rautou PE, Sarin S, Valla D, Paradis V, Moreau R. Diabetes mellitus in patients with cirrhosis: clinical implications and management. Liver Int. 2016;36(7):936-48. doi: 10.1111/liv.13115, PMID 26972930.

3.	Lee WG, Wells CI, McCall JL, Murphy R, Plank LD. Prevalence of diabetes in liver cirrhosis: A systematic review and meta-analysis. Diabetes Metab Res Rev. 2019;35(6):e3157. doi: 10.1002/dmrr.3157, PMID 30901133.”

**-L87-91,** please support your hypothesis with references;

**Response:** We have added references to support our hypothesis and the rationale for using the single-leg stance test (SLST).

**(Introduction, Page 5, Lines 80–82)**

“Given that DM-related complications are known to impair balance and reduce SLST duration [17] [18] [19], the aim of this study was to examine whether comorbid DM is associated with impaired SLST in patients with CLD.”

**-in materials and methods section,** the order of evaluations and rest between them should be described;

**Response:** We have added a description of the evaluation order and confirmed that appropriate rest periods were provided between tests.

**(Methods, Page 6, Lines 110-116)**

“Patient characteristics and blood test data were evaluated at the time of hospital admission. Body composition and physical function were assessed at the initiation of physical therapy. The interval between admission and the start of physical therapy was less than three days. Body composition assessment was performed first, prior to the initial physical therapy session. Physical functions, such as SLST and muscle strength, were evaluated by physical therapists in the hospital rehabilitation room. Sufficient rest periods were provided between each test to ensure no interference with the results.”

-please add the type of the study;

**Response:** We have explicitly stated that this is a retrospective cross-sectional study in the Methods section.

**(Methods, Page 5, Lines 86)**

“This was a retrospective cross-sectional study.”

**-L140,** please describe the time of the day for the assessment and what were the conditions applied before the assessment;

**Response:** We have added information regarding the timing of assessments (conducted during daytime) and the conditions (e.g., before heavy exercise) in the Methods section.

**(Methods, Page 6, Lines 112-116)**

“The interval between admission and the start of physical therapy was less than three days. Body composition assessment was performed first, prior to the initial physical therapy session. Physical functions, such as SLST and muscle strength, were evaluated by physical therapists in the hospital rehabilitation room. Sufficient rest periods were provided between each test to ensure no interference with the results.”

**(Methods, Page 7, Lines 136-140)**

“Skeletal muscle mass was measured using bioelectrical impedance analysis (InBody720; InBody Co., Ltd., Seoul, Korea). The validity of this device for assessing muscle mass has been previously established in patients with CLD [8] [25]. To ensure data consistency, all measurements were conducted during the daytime, after voiding, and at least 2 hours after the last meal.”

**-L142,** please add information about validity of the device;

**Response:** We have added a statement and references regarding the validity and reliability of the bioelectrical impedance analysis device used in this study.

**(Methods, Page 7, Lines 137-140)**

“The validity of this device for assessing muscle mass has been previously established in patients with CLD [8] [25]. To ensure data consistency, all measurements were conducted during the daytime, after voiding, and at least 2 hours after the last meal.”

**(References)**

“8.	Nishikawa H, Shiraki M, Hiramatsu A, Hara N, Moriya K, Hino K, et al. Reduced handgrip strength predicts poorer survival in chronic liver diseases: a large multicenter study in Japan. Hepatol Res. 2021;51(9):957-67. doi: 10.1111/hepr.13679, PMID 34057800.

“25.	Nishikawa H, Shiraki M, Hiramatsu A, Moriya K, Hino K, Nishiguchi S. Japan Society of Hepatology guidelines for sarcopenia in liver disease. 1st ed: Recommendation from the working group for creation of sarcopenia assessment criteria. Hepatology Research. 2016;46(10):951-63.”

**-L206,** effect sizes should be calculated and added for the appropriate analysis;

**Response:** Effect sizes have been calculated and added to the Results section and Tables 2 and 3.

**(Methods, Page 10, Lines 207-217)**

“All continuous variables were first subjected to the Shapiro–Wilk test to assess normality. Variables that followed a normal distribution were presented as mean (± standard deviation) and were compared between groups using the Student’s t-test, with Cohen’s d calculated as the effect size. Variables that deviated from normality were expressed as median (interquartile range) and compared using the Mann–Whitney U test. For the U test, the effect size r was calculated using the formula r = |Z| / √N. Categorical variables were presented as absolute numbers and percentages and compared using the Fisher’s exact test or chi-squared test, as appropriate. The association for categorical variables was evaluated using Cramer’s *V* as the effect size. Effect sizes were interpreted using the following criteria: Cohen’s *d* (0.2–0.5 = small, 0.5–0.8 = medium, and ≥ 0.8 = large), *r*, and Cramer’s *V* (0.1–0.3 = small, 0.3–0.5 = medium, and ≥ 0.5 = large) [38].

**Reference**

38.	Cohen J. Statistical power analysis for the behavioral sciences. routledge; 2013. doi: 10.4324/9780203771587.

**(Methods, Page 11, Lines 222-223)**

“and the model fit was assessed using the Nagelkerke pseudo-*R*^2^.”

**(Results, Table 2, S2 Table, Table 3)**

**(Results, Page 19, 309)**

“with impaired SLST (Nagelkerke pseudo-*R^2^* 0.31, p<0.001).”

**-Table 1,** please check table format and size because it is too long (3 pages). In addition, please add units for all variables;

**Response:** We revised the format of Table 1 to improve readability and added units for all the variables. To reduce the lengths of **Tables 1** and **2,** detailed data were moved to the Supporting Information **(S1 Table and S2 Table)**.

**-for tables 2 and 3,** please add effect sizes and their interpretation

**Response:** Effect sizes were calculated for all analyses and added to **Tables 2, S2 Table,** and **Table 3**.

**(Results, Page 19, 309)**

“with impaired SLST (Nagelkerke pseudo-R2 0.31, p<0.001).”

To the Reviewer #3

Key Revisions Requested

**Abstract**

1. **Line 45:** "Patients with DM had a significantly higher prevalence of impaired SLST than those without DM (20% vs 0%, p = 0.002)." A 0% rate in the non-DM group is striking. While statistically significant, it is worth noting that the non-DM group was small (n = 34), and this result may be sensitive to sample size.

**Response:** We agree that the 0% prevalence in the non-DM group is striking and likely influenced by the small sample size of that group (n = 34). We have added a comment regarding this as a limitation in the Discussion section.

**(Discussion, Page 25, Lines 440-443)**

“Ninth, the non-DM group had a relatively small sample size (n = 34) with a 0% incidence of impaired SLST. This lack of events in the control group may lead to unstable estimates in the comparative analysis, which should be considered when interpreting the results.”

**Introduction**

1. **Lines 87-91:** The hypothesis and aim are clear. Well-stated.

**Methods**

1. **Timing of Assessments (Lines 95-103, 118-121):** The protocol where blood tests (admission) and physical function assessments (start of physiotherapy) occurred at different, unspecified times is a significant flaw. Clinical status, especially fluid balance (ECW/TBW), can change rapidly, introducing substantial measurement bias and weakening the core cross-sectional associations.

**Response:**

We fully acknowledge the reviewer’s concern that blood tests and physical function assessments were not conducted simultaneously, which represents a potential source of measurement bias. In particular, clinical status—including fluid balance as reflected by ECW/TBW—can change over a short period of time, potentially weakening cross-sectional associations.

To address this concern and improve transparency, we have clarified the timing and sequence of all assessments in the Methods section. Specifically, we now state that body composition and physical function were assessed within three days of admission and that body composition measurements were performed prior to the initiation of physical therapy. In addition, all measurements were conducted during the daytime and at least two hours after the last meal to enhance data consistency **(Methods, Page 6, Lines 110–116 and Page 7, 136–140).**

“Patient characteristics and blood test data were evaluated at the time of hospital admission. Body composition and physical function were assessed at the initiation of physical therapy. The interval between admission and the start of physical therapy was less than three days. Body composition assessment was performed first, prior to the initial physical therapy session. Physical functions, such as SLST and muscle strength, were evaluated by physical therapists in the hospital rehabilitation room. Sufficient rest periods were provided between each test to ensure no interference with the results.”

“Skeletal muscle mass was measured using bioelectrical impedance analysis (InBody720; InBody Co., Ltd., Seoul, Korea). The validity of this device for assessing muscle mass has been previously established in patients with CLD [8] [25]. To ensure data consistency, all measurements were conducted during the daytime, after voiding, and at least 2 hours after the last meal.”

**(References)**

“8.	Nishikawa H, Shiraki M, Hiramatsu A, Hara N, Moriya K, Hino K, et al. Reduced handgrip strength predicts poorer survival in chronic liver diseases: a large multicenter study in Japan. Hepatol Res. 2021;51(9):957-67. doi: 10.1111/hepr.13679, PMID 34057800.

“25.	Nishikawa H, Shiraki M, Hiramatsu A, Moriya K, Hino K, Nishiguchi S. Japan Society of Hepatology guidelines for sarcopenia in liver disease. 1st ed: Recommendation from the working group for creation of sarcopenia assessment criteria. Hepatology Research. 2016;46(10):951-63.”

Importantly, although we acknowledge that changes in fluid balance during this short interval cannot be completely excluded, the assessment window was intentionally restricted to less than three days, and all measurements were performed before any structured physical therapy intervention was initiated. This standardized protocol was intended to minimize systematic bias related to treatment effects.

Nevertheless, we recognize that ECW/TBW is particularly sensitive to short-term fluctuations, and therefore we have explicitly stated this issue as a study limitation in the Discussion section. We now emphasize that differences in assessment timing may have introduced measurement bias and that this limitation should be considered when interpreting the results.

**(Discussion, Pages 24–25, Lines 421–425).**

“Second, blood tests were performed at admission, whereas body composition and physical function were assessed within three days after admission. Changes in clinical status during this interval, including alterations in fluid balance, may have introduced measurement bias, particularly for variables such as ECW/TBW that are sensitive to short-term fluctuations.”

2. **Unmeasured Confounding - Diabetic Neuropathy (Throughout):** The most critical limitation is the absence of any assessment or adjustment for diabetic peripheral neuropathy. As a primary mechanism for balance impairment in DM, neuropathy is a profound confounder. The observed association between DM and impaired SLST may be entirely mediated by neuropathy, drastically altering the interpretation of the results.

**Response:** This is a crucial point. As we did not objectively assess diabetic peripheral neuropathy, it remains a major unmeasured confounder. We have now explicitly acknowledged this in the Discussion, stating that the association between DM and impaired SLST might be mediated by neuropathy.

**(Discussion, Page 25, Lines 427-432)**

“Fourth, a major limitation of this study is the lack of assessment for diabetic peripheral neuropathy. As neuropathy is a primary mechanism for balance impairment in DM, it represents a significant unmeasured confounder. Therefore, we cannot exclude the possibility that the observed association between DM and impaired SLST is mediated, at least in part, by neuropathy rather than fluid distribution alone. Fifth, detailed information regarding the duration of DM, specific medications, and history of falls was not available.”

3. **Sample Generalizability (Lines 232-236):** The cohort, with a remarkably high DM prevalence (78%) and predominantly compensated liver disease (83% Child-Pugh A), is highly selective. This limits the generalizability of the findings to the broader CLD population and should be explicitly acknowledged as a limitation.

**Response:** We agree that our cohort is highly selective (78% DM prevalence, 83% Child-Pugh A). We have added a statement acknowledging that these characteristics may limit the generalizability of our findings to the broader CLD population.

**(Discussion, Page 25, Lines 436-440)**

. Eighth, our study population had a remarkably high prevalence of DM (78%) and predominantly comprised patients with compensated liver disease (83% Child-Pugh A). This high selectivity may limit the generalizability of our findings to the broader CLD population, and the possibility of selection bias cannot be excluded. ”

**Results**

1. **Lines 272-279:** When presenting comparative results (e.g., KEF/weight, grip strength), ensure that the descriptive statistics (mean ± SD vs. median [IQR]) match the tests used (t-test vs. Mann-Whitney U) as reported in the tables.

**Response:** We have carefully reviewed the Results section and ensured that the presentation of mean ± SD (for t-tests) and median [IQR] (for Mann–Whitney U tests) is consistent with the statistical methods used.

**Discussion**

1. **Lines 368-371:** The discussion about the null finding for ammonia is reasonable given the population's characteristics.

2. **Lines 392-394, 441-443:** The concept of "subclinical edema" is interesting and a useful clinical takeaway. This is well-argued.

3. **Lines 395-419:** The pathophysiological discussion is comprehensive but somewhat speculative, especially the detailed molecular mechanisms (IR/IGF-IR/FoxO, myostatin), given that the study did not measure any of these parameters. This section could be shortened and framed more as potential explanatory hypotheses for future research rather than established explanations for the current findings.

**Response:** Following the reviewer’s suggestion, we have significantly shortened the discussion regarding molecular mechanisms (IR/IGF-IR/FoxO, myostatin). We have reframed these points as potential explanatory hypotheses for future research rather than established conclusions of the present study.

**(Discussion, Page 24, Lines 407-418)**

“The association between increased ECW/TBW and impaired SLST in patients with CLD and comorbid DM likely involves multiple pathophysiological mechanisms. In liver cirrhosis, portal hypertension and lymphatic dysfunction lead to interstitial fluid accumulation and lower extremity edema [5] [52], which may impair muscle strength and proprioception, thereby diminishing balance [53, 54]. Furthermore, insulin resistance—a hallmark of both CLD and DM—may activate proteasome and autophagy pathways via impaired signaling, potentially leading to muscle wasting and weakness [55] [56]. Additionally, elevated serum myostatin levels in progressive CLD may suppress muscle protein synthesis [57] [58]. Collectively, although the exact pathways remain unclear, changes in interstitial fluid dynamics, insulin resistance-induced signaling impairment, and disrupted liver–muscle crosstalk are potential factors linking increased ECW/TBW with impaired SLST.”

References:

5.	García-Compeán D, Orsi E, Kumar R, Gundling F, Nishida T, Villarreal-Pérez JZ, et al. Clinical implications of diabetes in chronic liver disease: diagnosis, outcomes and management, current and future perspectives. World J Gastroenterol. 2022;28(8):775-93. doi: 10.3748/wjg.v28.i8.775, PMID 35317103.

52.	Kumar R, Anand U, Priyadarshi RN. Lymphatic dysfunction in advanced cirrhosis: contextual perspective and clinical implications. World J Hepatol. 2021;13(3):300-14. doi: 10.4254/wjh.v13.i3.300, PMID 33815674.

55.	Barzilay JI, Cotsonis GA, Walston J, Schwartz AV, Satterfield S, Miljkovic I, et al. Insulin resistance is associated with decreased quadriceps muscle strength in nondiabetic adults aged >or=70 years. Diabetes Care. 2009;32(4):736-8. doi: 10.2337/dc08-1781, PMID 19171728.

56.	O’Neill BT, Lee KY, Klaus K, Softic S, Krumpoch MT, Fentz J, et al. Insulin and IGF-1 receptors regulate FoxO-mediated signaling in muscle proteostasis. J Clin Invest. 2016;126(9):3433-46. doi: 10.1172/jci86522, PMID 27525440.

57.	Kirk B, Feehan J, Lombardi G, Duque G. Muscle, bone, and fat crosstalk: the biological role of myokines, osteokines, and adipokines. Curr Osteoporos Rep. 2020;18(4):388-400. doi: 10.1007/s11914-020-00599-y, PMID 32529456.

58.	Nishikawa H, Enomoto H, Ishii A, Iwata Y, Miyamoto Y, Ishii N, et al. Elevated serum myostatin level is associated with worse survival in patients with liver cirrhosis. J Cachexia Sarcopenia Muscle. 2017;8(6):915-25. doi: 10.1002/jcsm.12212, PMID 28627027.

**Conclusion**

1. The conclusions are generally supported by the data but should be tempered by acknowledging the key limitations, such as the cross-sectional design, lack of data on neuropathy, and the specific characteristics of the study cohort.

**Response:** We have revised the Conclusion to be more tempered, explicitly mentioning that findings should be interpreted considering limitations such as the cross-sectional design and the lack of neuropathy data.

**(Discussion, Pages 24-26, Lines 419-446)**

“This study has several limitations. First, due to its cross-sectional design, causal relationships cannot be established. Longitudinal prospective studies are required to further elucidate these associations. Second, blood tests were performed at admission, whereas body composition and physical function were assessed within three days after admission. Changes in clinical status during this interval, including alterations in fluid balance, may have introduced measurement bias, particularly for variables such as ECW/TBW that are sensitive to short-term fluctuations. Third, distinguishing between type 2 DM and hepatogenous DM in patients with CLD remains difficult, as noted in previous studies involving similar populations. Fourth, a major limitation of this study is the lack of assessment for diabetic peripheral neuropathy. As neuropathy is a primary mechanism for balance impairment in DM, it represents a significant unmeasured confounder. Therefore, we cannot exclude the possibility that the observed association between DM and impaired SLST is mediated, at least in part, by neuropathy rather than fluid distribution alone. Fifth, detailed information regarding the duration of DM, specific medications, and history of falls was not available. Sixth, the small sample sizes in the HBV, ALD, and other etiology groups limited our ability to detect significant differences between subgroups. Seventh, the limited variability in NH₃ levels among our participants, reflecting predominantly mild CLD, may have obscured any association with SLST results. Eighth, our study population had a remarkably high prevalence of DM (78%) and predominantly comprised patients with compensated liver disease (83% Child-Pugh A). This high selectivity may limit the generalizability of our findings to the broader CLD population, and the possibility of selection bias cannot be excluded. Ninth, the non-DM group had a relatively small sample size (n = 34) with a 0% incidence of impaired SLST. This lack of events in the control group may lead to unstable estimates in the comparative analysis, which should be considered when interpreting the results. Finally, the ECW/TBW cutoff value of 0.393 was derived from the present dataset, and external validation is needed prior to clinical application. Future prospective studies should aim to collect more detailed data on these variables to validate and expand upon our findings.”

**(Conclusions, Page 25, Lines 449-460)**

“DM was associated with a higher prevalence of impaired SLST in patients with CLD. Age, BMI, and ECW/TBW were identified as significant factors associated with impaired SLST. These findings suggest that SLST may be a useful tool for routine assessments of balance in patients with CLD and comorbid DM. Exercise therapy aimed at weight reduction may also contribute to improvements in balance. Furthermore, an ECW/TBW of 0.393 was identified as a potential cutoff for predicting impaired SLST. From a fall-prevention perspective, even mild overhydration (ECW/TBW 0.390–0.400) may warrant attention in patients with CLD and comorbid DM.

However, these findings should be interpreted cautiously given the cross-sectional design of the study, the lack of objective assessment of diabetic peripheral neuropathy, and potential measurement bias related to differences in assessment timing. Further prospective studies are required to confirm these associations and clarify their clinical implications.”
